# Transforming healthcare through in-body bioelectronic systems

**DOI:** 10.1038/s41467-026-71188-3

**Published:** 2026-04-11

**Authors:** Steven Ceto, Stacey Amanda Elshove, Mingzheng Wu, Khalil Ramadi, Christoph Tondera, Ivan Rusev Minev, Shriya Srinivasan, Kyuhwa Lee, Michalina J. Gora, John Rogers, Claudia Kathe, Thomas Haynes Hutson

**Affiliations:** 1 Wyss Center for Bio and Neuroengineering, Geneva, Switzerland; 2 Department of Fundamental Neurosciences, University of Lausanne (UNIL), Lausanne, Switzerland; 3 Querrey Simpson Institute for Bioelectronics, Northwestern University, Evanston, IL USA; 4 Division of Engineering, New York University Abu Dhabi, Abu Dhabi, UAE; 5 Tandon School of Engineering, New York University, New York, NY USA; 6 Institute of Biofunctional Polymer Materials, Leibniz Institute of Polymer Research Dresden, Dresden, Germany; 7 Center for Regenerative Therapies Dresden, TUD Dresden University of Technology, Dresden, Germany; 8 Else Kröner Fresenius Center for Digital Health, Medical Faculty Carl Gustav Carus, TUD Dresden University of Technology, Dresden, Germany; 9 Division of Gastroenterology, Hepatology and Endoscopy, Brigham and Women’s Hospital, Harvard Medical School, Boston, MA USA; 10 David H. Koch Institute for Integrative Cancer Research, Massachusetts Institute of Technology, Cambridge, MA USA; 11 ICube Laboratory, UMR7357 CNRS University of Strasbourg, Strasbourg, France

**Keywords:** Therapeutics, Biomedical engineering, Biomaterials, Translational research, Gene therapy

## Abstract

Recent advances in the development of in-body bioelectronic systems are providing new opportunities for the clinical management of various diseases and disorders. These emerging technologies are tailored to specific organs and are beginning to blend both diagnostic sensing and therapeutic actuation. The aim of these systems is to seamlessly integrate with the physiological environment, as illustrated by the diverse device strategies discussed throughout this article. Next generation modalities, such as optogenetics combining gene therapy with devices for photostimulation, are gaining popularity and offer advantages over existing therapeutic strategies. In this perspective, we explore the current state of technological developments, key challenges in the field and potential pathways for translating these innovations into clinical practice.

## Introduction

**Fig. 1:**
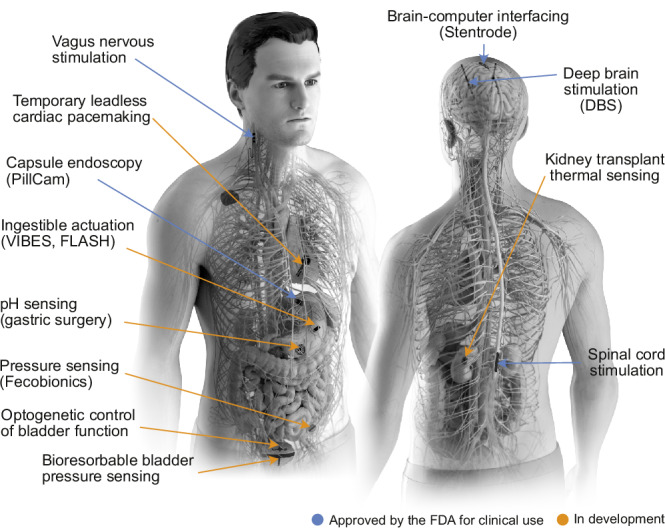
Bioelectronic devices target diverse organ functions. The technologies highlighted in this Perspective are summarized here and grouped according to their status: either already in clinical use or still in development, with many having undergone extensive testing in preclinical models.

Until recently implantable technologies were primarily designed as single-mode actuators, with electrical stimulation being one of the most prominent examples used to achieve a therapeutic effect. Biological sensing capabilities on the other hand were either indirect, highly invasive, or dependent on bulky external equipment. The integration of diagnostic sensing and therapeutic actuation within a single system was rare. As the field continued to advance, modern bioelectronic systems have evolved to address more complex demands for monitoring and managing diverse types of organ functions, offering transformative engineering-based opportunities that complement or replace traditional surgical, early bioengineering and pharmaceutical approaches^1^ (Fig. 1). The development of these systems is guided by the anatomy and physiological processes of the target organs, which influences the selection of sensing modalities, actuation strategies, and operational timeframes. Despite targeting different organ systems, many of these technologies share common features and challenges. Specific in-depth reviews on various technologies comprehensively showcase the field’s recent advances (non-exhaustive list^2–7^); here, we provide a focused perspective that highlights select innovations, current challenges, and future opportunities for in-body bioelectronic technologies. We specifically discuss emerging materials for bioelectronics, such as hydrogels, that facilitate integration with soft and complex tissues, while creating new possibilities to provide actuation and/or sensing. We explore implantable technologies that are bioresorbable and capable of monitoring organ-specific physiological functions, as well as ingestible bioelectronics and fecobionics, which offer indirect organ integration and present unique challenges. We discuss neuromodulation technologies, highlighting how novel modalities, such as optogenetic technologies, offer a potential for greater precision and specificity to target various neuronal functions compared to traditional electrical stimulation. Finally, we address the future challenges facing the field of in-body bioelectronics. We discuss how integrating closed-loop control and artificial intelligence (AI) are starting to be implemented to enhance the performance of these systems and the adaptability for individual patient needs, ultimately improving patient outcomes and reshaping disease management strategies.

## Advanced materials for implantable bioelectronic technologies

**Fig. 2:**
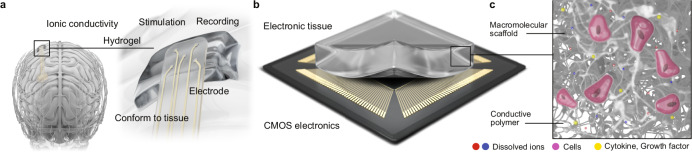
Conductive hydrogels as electronic tissues bridging the gap between electronics and biological systems. **a** The combination of mixed electronic and ionic conductivity makes conductive hydrogels promising for bioelectronic applications, as they may enable conformal tissue-device interfaces, enhanced stimulation and recording and even multimodal information exchange in the biomolecular domain. **b** Unlike stiff, metal-based CMOS electronics, electrically conductive hydrogels are soft, flexible and water-based. **c**. Their tuneable properties support interactions with cell instructive macromolecules such as cytokines, growth factors and cell adhesion proteins, enabling them to mimic some of the key functions of the extracellular matrix.

Conventional electronic implants rely on circuits sealed in rigid, bulky hermetic enclosures, with hard-wired tissue interfaces based on small numbers of macroscopic electrodes for electrical stimulation or electrophysiology^8^. Emerging materials and device architectures are enabling flexible, stretchable bioelectronic interfaces that better integrate with dynamic three-dimensional tissues while expanding implanted systems beyond traditional prosthetics toward broader therapeutic and tissue-repair applications^9–11^ (Fig. 2a).

A promising class of materials are electrically conductive hydrogels. Hydrogels are crosslinked networks of hydrophilic polymers swollen with water and are well-known biomaterials for tissue engineering^12^. In the specific case of conductive hydrogels, they are engineered to transport electric charge by both ions and electrons (dual electronic-ionic conductivity). This can be achieved by incorporating a percolating network of inorganic conductive particles (e.g., metallic nanowires) in the hydrogel network. Alternatively, organic semiconductive polymers such as Poly(3,4-ethylenedioxythiophene) (PEDOT), Polyaniline (PANI), their derivatives as well as other conjugated polymers can be used to form an interpenetrating network in the hydrogel^13^. In the context of implantable bioelectronic devices, conductive hydrogels could address the soft-to-rigid mismatch encountered with implanted bioelectronics because they are mostly made of water (typically > 80%) and their viscoelastic properties can be engineered to mimic those of tissues^13^. Conductive hydrogels are already impacting the design of electrode arrays for stimulation and recording. In addition to mechanical softness, due to their three-dimensional nanoarchitecture (volumetric capacitance), hydrogel based electrodes can reach higher charge injection capacities than metal electrodes with similar dimensions^14^. They can also act as reservoirs for therapeutic small molecules^15^, applying external potential can change the redox state of the semiconductive polymer component and lead to electrically controlled release of the molecular cargo^16^. A similar effect has been exploited in mechanical actuators where swelling can be electrically controlled causing electrodes to wrap around peripheral nerves on command^17^. Conductive hydrogels have been integrated in organic electrochemical transistors (OECTs), as the channel whose transconductance can be switched by ions and molecules in the surrounding electrolyte^18,19^. They can render this architecture optimal for sensing weak bioelectronic signals and low concentrations of soluble biomarkers^20^. Hydrogel based OECTs can also form elements with non-linear electrical response for applications in reservoir computing^21^. Studies have shown that semiconductive polymers can be formed in the space between living cells, essentially creating conductive hydrogels in situ in tissue using the native extracellular matrix as a template^22,23^.

Many opportunities and challenges remain before the advantages of conductive hydrogels can be fully exploited in implantable bioelectronic systems. One promising strategy is to build conductive hydrogels from macromolecules already present in the extracellular matrix (collagens, elastin, proteoglycans, and glycosaminoglycans^24^) and synthetic semiconductive polymers^25,26^. This may result in materials that combine electroactivity and bioactivity. In the long term, this may lead to a new class of biomaterials that we have termed “electronic tissues” in which electrical inputs are directly converted into biomolecular signals enabling electrotaxis, remodeling of native extracellular matrices, sequestration and release of growth factors, cytokines, and morphogens. These “electronic tissues” could couple biological processes directly to conventional Complementary Metal Oxide Semiconductor (CMOS) electronics as well as to emerging neuromorphic and reservoir computing systems (Fig. 2b–c). A separate set of challenges concerns processing conductive hydrogels into devices^27^. One key issue is achieving reliable patterning at micro- and nanometer resolution while scaling arrays to hundreds and thousands of devices. Emerging printing technologies based on optical, electrochemical, and direct-deposition techniques are compatible with the hydrated state of the materials and may even be applied inside living tissues. A key enabler will be the development of inks of oligomeric and macromolecular building blocks that can be assembled using orthogonal chemistries. Fabricating fully hydrogel-based devices also requires suitable encapsulation materials to serve as the analogs of silicone, parylene or polyimide in conventional electrode arrays. Suitable materials will have to be electrically insulating, soft, bioinspired, potentially hydrated. Promising candidates include hydrogels with selective ion permeability and nanostructured lipid bilayers^28,29^. At present, most conductive hydrogels incorporating organic semiconductors rely on p-type polymers. With the development of stable n-type organic materials (e.g., poly(benzodifurandione) PBFDO), it may soon be possible to create hydrogels incorporating both p- and n-type polymer regions, enabling complementary logic circuits based on tissue-like materials^30,31^. Another open question is how to integrate conductive hydrogels with mechanically dissimilar materials, such as connectors to standard electronics. Here, it is essential to maintain both electrochemical stability (e.g., prevent corrosion of metal pads) and mechanical integrity to avoid delamination due to strain concentration at interfaces. Finally, the mode of introducing conductive hydrogel devices into the body is crucial. Minimally invasive strategies such as injection are particularly attractive. These approaches will require devices that can either tolerate large strains during extrusion or self-assemble from smaller building blocks once they reach their target location in the tissue.

Despite the many demonstrated applications and concepts for conductive hydrogels in academic research, substantial challenges remain before clinical translation is possible. The conductive phase of these hydrogels is non-native and may trigger immune responses or foreign-body reactions. Even when the native extracellular matrix is used as inspiration, the conductive polymers may need chemical modification to enhance biocompatibility, or replacement with protein-based conductive polymers, to reduce adverse responses^32^. It is also important to distinguish between hydrogel-based bioelectronics intended for chronic implantation and temporary devices designed to deliver a short-term therapeutic program before disassembly. In both cases, systematic studies are needed to evaluate the biological effects of degradation products, which may be monomers, oligomers or larger fragments. Equally crucial is improving the long-term stability of these materials, including resistance to degradation of electrical properties, for example, through overoxidation during use. Despite these constraints, devices incorporating conductive hydrogels have already shown promising in vitro and in vivo performance^33^. Neuro-hydrogel electrodes have been used to interface functionally and mechanically with soft tissues such as the brain, spinal cord, and peripheral nerves in a biomimetic manner^34^. Other applications, including multimodal stimulation to promote wound healing and simultaneous sensing to detect early-stage bacterial infection^35^, highlight the potential of these materials beyond classical electroactive tissue interfaces. Material technologies such as these can incorporate diverse electronic, optoelectronic, and microfluidic components, or even living cells (Box 1), engineered to interact with organs through spatiotemporally orchestrated electrical, optical, thermal, and pharmacological modalities^36^, as discussed in the next sections.

Box 1 Biohybrid neural interfaces Taking biocompatibility a step further, biohybrid neural interfaces seek to utilize living cells as the electrode interface with the target tissue^130,131^. The term “biohybrid” was first applied to insulin secretion devices seeded with islets in the mid-1980s^132^, and its application in neural interfaces began a decade later with the peripheral nerve. Interfacing with transected peripheral nerves has been achieved by growing axons from a biohybrid device directly into the distal nerve stump^131^, as well as through muscle cells^133^ or tissue^134^. To adapt the biohybrid approach to the central nervous system, Science Corporation has proposed a unique strategy in which neurons genetically modified to express light-sensitive opsins are seeded into wells of a device that contains microLEDs for optogenetic stimulation as well as standard recording electrodes (https://science.xyz/technologies/biohybrid/). When placed on the surface of the brain, the axons and dendrites of the seeded neurons extend into the cortex and synapse with neurons in the grey matter. This, in theory, dramatically reduces tissue damage and enables much higher electrode density than standard inorganic approaches. While no data has been published with the full device, preliminary studies with embryonic neurons loaded into a microwell scaffold and implanted on the cortical surface has proved promising^135^. Other biohybrid approaches in the central nervous system include coating electrodes with neurons prior to implantation^136,137^ and “living electrodes” comprising neurons with pre-elongated axons shuttled to the brain via microinjected hydrogels^138^. These methods would cause more insertional damage than allowing axons to grow into tissue but could still improve long-term biocompatibility of the implanted electrodes.

## Sensing and modulation of organ function

Many recent non-invasive, wearable technologies have demonstrated their ability to continuously measure and monitor physiological parameters, such as blood pressure and flow, temperature, blood glucose as well as interstitial fluid^37–39^. However, one of the limitations of these technologies is that they are restricted to monitoring superficial biological tissues, which limits their effectiveness in assessing deep tissue and timely detection of altered homeostasis. Similarly, non-invasive technologies for stimulation face challenges in precisely targeting deep tissues and organs without affecting superficial tissues, resulting in reduced specificity as they attempt deeper penetration. While traditional modalities, like electrical stimulation, can be optimized to improve targeting and specificity by considering the different characteristics of tissues and organs, these improvements remain generally limited.

Recent implantable bioelectronic systems offer promising alternatives with superior specificity and speed of detection compared to non-invasive technologies. These systems can integrate multiple modalities to achieve more precise monitoring and interventions in deep tissues. Examples include biosensors capable of measuring local organ temperature and thermal conductivity, which serve as biophysical surrogates for inflammation and tissue perfusion. Devices with such capabilities have been used to enable early detection of kidney transplant rejection^40^ (Fig. 1). In such cases, a soft thermal sensor is directly mounted on the kidney’s surface and connects to a wireless electronics module secured to the adjacent abdominal wall. This module contains sensor readout circuitry and Bluetooth low-energy system-on-chip (BLE SoC) for control and data transmission, all powered by a coin-cell battery. Similar technologies have been developed for long-term continuous monitoring through temperature sensors of inflammatory bowel diseases^41^, and hemodynamic measurements in patients at risk of cardiovascular diseases performed by a device consisting of sensing modules for blood pressure, flow and temperature inserted into blood vessels^42^.

When organ monitoring is required only for a limited time, such as after surgical interventions, temporary, bioresorbable and wireless monitoring strategies become highly attractive. These biosensors are engineered to detect environmental changes, often in deep tissues, and then gradually dissolve, eliminating the need for surgical removal. They commonly incorporate bioresorbable metals^43^ and hydrogels^44–47^ that break down through dissolution reactions and hydrolytic chain scissions when exposed to biofluids, transforming into harmless byproducts over controlled time periods. An example of a bioresorbable device is a hydrogel sensor that swells in response to pH changes, with different chemical compositions allowing them to operate across specific pH ranges. This swelling leads to conformational changes of embedded metal components, which are also bioresorbable and can be monitored using ultrasound. Such sensors can temporarily detect physiological conditions such as acidic environments that might indicate gastric leaks after a laparoscopic sleeve gastrectomy^48^ (Fig. 1), or alkaline conditions related to pancreatic disorders^48^.

Integrating electronics into bioresorbable devices serves to allow more sophisticated and continuous monitoring, rather than relying on regular monitoring by, for instance, ultrasound examination. This has been demonstrated for pH sensing after gastric sleeve surgery, where inductor-capacitor resonant circuits efficiently convert dimensional changes into shifts in resonant frequencies, with magnitudes that can be quantified accurately by inductive coupling to an external reader device^49^. The material selection and design features were selected so that the device disintegrates and naturally dissolves, in a biocompatible fashion without residue after the post-surgical recovery period has elapsed, thereby eliminating the risks and costs associated with an additional surgery for extraction. Another example of bioresorbable bioelectronic devices include pressure sensors to measure bladder pressure after cystectomies^50^ and actuators such as thin, flexible and leadless temporary cardiac pacemakers^51^ that can provide the necessary electrotherapy (Fig. 1). Devices such as these can be implemented as temporary solutions for monitoring following surgical interventions, or to assess whether more permanent solutions are needed. In ideal scenarios, these devices are linked to wireless and minimally invasive platforms that support high-bandwidth, two-way communication and power transfer^52^, allowing for real-time programmable manipulation in response to biological feedback^53,54^. Collectively, they provide precise diagnostic and therapeutic capabilities for early detection, timely management, and personalized treatment to improve patient outcomes. More importantly, they can potentially aid in patient stratification, helping to identify and exclude non-responders to modulation therapies with chronic implants.

A limitation to the clinical translation of bioresorbable implants is that regulatory agencies currently lack established frameworks for electronic implants designed to dissolve intentionally^55^. Developers must demonstrate that each constituent material degrades into nontoxic byproducts that can be fully and naturally eliminated from the body^56^. To date, clinical use has been limited to select cases. For example, the US Food and Drug Administration (FDA) authorized a sutureless nerve repair implant (DEN240066) composed of biodegradable polymers in 2025, marking one of the first clearances for a resorbable device^57^. However, clear guidelines regarding device lifespan, degradation rate, and end-of-life verification remain absent, making regulatory approvals a case-by-case learning process^58^.

Manufacturing and scalability pose additional challenges. Many transient electronic devices depend on unconventional materials outside standard semiconductor fabrication. Producing clinically reliable devices from these materials at scale, with consistent dissolution rates and functional performance, remains a substantial hurdle. High costs, limited industrial-scale production infrastructure and the need to balance long-term storage stability with precise, on-demand post-implantation degradation add further complexity^56^. Moreover, integrating transient components with conventional electronics (e.g., for wireless power or control) requires hybrid manufacturing strategies, as only portions of the system are bioresorbable. Each of these obstacles must be addressed to transition bioresorbable electronics from laboratory prototypes to practical, mass-producible medical devices.

Focusing on short-term therapeutic applications where transient implants offer clear advantages over permanent devices is a promising strategy for clinical translation. Bioresorbable nerve stimulators illustrate this approach, demonstrating weeks-to-months functionality in animal models and matching clinically relevant treatment windows^59^, particularly for post-operative recovery or rehabilitation. In cardiovascular care, bioabsorbable stents and scaffolds^60^ are re-emerging with improved designs following earlier setbacks, providing temporary vascular support during healing while avoiding long-term complications. Therefore, we propose that initial clinical trials should focus on narrowly defined indications, including temporary cardiac pacing^61^, biodegradable brain interfaces for acute injury^62^, or transient stimulators for post-surgical pain management^63^. As materials science improves the predictability and scalability of biodegradable components, bioresorbable electronics will move toward wider clinical adoption.

## From ingestible devices to fecobionics

In contrast to implantable devices, another emerging class of bioelectronics for interfacing with the body are ingestible devices, which leverage the gastrointestinal (GI) lumen being ‘outside the body’, imposing distinct design considerations. By directly contacting the GI tract, ingestibles provide minimally invasive means to monitor, diagnose, and treat the organ systems connected to the GI tract, including the liver, pancreas, biliary system, brain, and systemic circulation. Beyond nutrient absorption, the gut’s role in metabolic regulation is not only mediated by endocrine pathways, but also tightly linked to the enteric and central nervous system via vagal and spinal pathways^64^. As such, ingestible devices that interface with enteric neurons can exert widespread physiological effects. Additionally, the gut’s microbiome, virome, and mycobiome (i.e., bacteria, viruses, and fungi), significantly affect physiology and immunity, offering additional therapeutic and diagnostic opportunities.

The first ingestible device was reported in 1957, a swallowable radio^65^. Subsequently, the development of capsule endoscopy systems laid the groundwork for ingestible devices, defining safe sizes for ingestion, onboard housing of active electronics, and materials durable enough for the harsh GI tract environment^66^. While fibrosis is not a major concern, devices rather must resist fouling due to the chemically harsh luminal environment, engage reliably with the mucosal surface, withstand cyclic expansion and contraction of the lumen and have surface geometries that don’t present a risk of perforation or obstruction^67^. While the pill format is commonly utilized, alternative geometries have been developed for gastric resident systems^68^, luminal contact^69^, and actuation^70^ functionalities. The design of these devices must factor in considerations of passage, degradation, propulsion, orientation, and interaction with sphincters.

The majority of currently developed ingestible devices for actuation fall within three major categories: chemical, electrical and mechanical actuation. Devices performing chemical actuation for drug delivery have evolved from simple dissolvable tablets, to coated capsules dissolving in specific pH environments, to actuated mechanisms for release of unstable drugs in target areas of the tract. Recent platforms enable delivery of macromolecules and biologics, such as insulin using self-deploying needles and self-orienting systems^71,72^, and have mucus-clearing surface features to improve drug absorption^73^. Combining electronic systems with drug delivery platforms can improve the uptake and reliability of delivered substances by locally increasing absorption and by having onboard reservoirs to prevent drug degradation^74^, expanding the range of therapeutics that can be delivered. Next, electrical actuation offers precise spatiotemporal control of stimulation targetable to specific locations of the tissue and can be turned on/off on-demand via external or onboard controllers. Electrical stimulation can specifically activate neurons engaging the gut-brain axis and other electrically-excitable cells, such as muscles^70,75^. The Fluid-wicking capsule for Active Stimulation and Hormone modulation (FLASH, Fig. 1), for example, uses electrical stimulation of the stomach mucosa to regulate hunger circuits^69^. Lastly, mechanical actuation exploits the gut’s mechanosensation. The Vibrating Ingestible BioElectronic Stimulator (VIBES, Fig. 1) is a device using vibration to activate gastric stretch receptors and thereby engage satiety and metabolic signaling circuits^76^. Similarly, a vibrating capsule by Vibrant Ltd. has been shown to improve bowel symptoms in patients with chronic constipation^77^. Other devices have been designed for mechanical capture for biopsy^78^.

Ingestible devices for sensing can detect motility, device location, vital signs, and chemical or biologic markers. Continuous monitoring of physiological parameters allows correlation with disease pathologies using large-scale data models. Several recent devices have detected gastric motility and the activity of nearby organs, such as the lungs and heart^79–81^. Other devices have been developed to sense intestinal gases^82^, biochemical markers^83^, and the microbiome^84–86^, readouts that can be used as indicators of diseases.

In the future, ingestible devices can be improved by increasing the stability, powering and specificity of luminal sensing. Devices should evolve to allow extended residence in the gut to monitor various elements of health to yield deeper insights of our health. There may be significant utility in combining sensing and actuation mechanisms for closed-loop control of physiology, enabling greater temporal and spatial targeting which reduces off-target effects. For example, an ingestible device could sense areas of pre-cancerous lesions or reduced mobility throughout the digestive system and locally deliver drugs or electromechanical stimulation. This may be especially useful in neurodegenerative disorders such as Parkinson’s or Alzheimer’s disease^87^, where gut biomarkers predate peripheral symptoms. Such a closed-loop therapeutic approach could mimic the success of existing closed-loop neuromodulation systems. For instance, the clinical success of vagus nerve stimulation to treat autoimmune conditions suggest that such neuromodulation might also be delivered through ingestible devices that activate vagal afferents present in the gut^88^. Devices could also be used to interface and modulate the microbiome to treat dysbiosis in conditions such as small intestinal bacterial overgrowth (SIBO). Ingestible platforms will have to incorporate advances in power management (either through power harvesting or wireless powering), remote communication, and mechanisms for extended GI retention. Closed-loop control may also require tracking of device location within the GI tract^89^. Future bioelectronic devices could also be designed to come in the form of edible electronics made of food materials^90^, and degrade before excretion to minimize risk of obstruction.

To date, most clinical applications of ingestible devices have focused on the stomach, where access is easy and risk of obstruction low. Translation to the small and large intestines remains challenging. Newer strategies, such as magnetic steering and active locomotion^91^, or thinly tethered devices aim to extend access to distal regions of the gut. One such example is a tethered capsule optical coherence tomography device for early screening of lesions in the oesophagus and duodenum^92^. However, these approaches still do not generally extend to the colon, thus leaving the clinical need for new sensing and modulation solutions in the lower digestive system, where inflammatory and functional disorders are common. Fecobionics, as a multimodal luminal device comprising sensors for pressure measurement and geometrical mapping embedded within a compliant soft design to mimic normal physiology, has been proposed to address the need of minimally invasive measurement of lower digestive system function^93^ (Fig. 1). Successful results from the first clinical trial of this multimodal bioelectronic device have shown potential to assess anorectal dysfunctions and open new opportunities for biofeedback training to alleviate dysfunction. To further understand the physiological changes underlying disorders of gut-brain interaction, new endoscopic approaches for measuring colonic electrophysiology have recently been developed^94,95^. Soft electrodes and hydrogels are one method which could be used to maintain contact between electrode surfaces and the dynamically moving colonic tissue^95^.

The design of future ingestible and luminal bioelectronics should consider specific clinical use cases and be co-developed with clinicians to inform functionality and utility. Clinical translation of new ingestible devices will be accelerated by FDA-approved predicates such as the SmartPill (K092342) and PillCam (K211684, Fig. 1). If translation is driven by local conditions in the gut, the challenges of clinical applications are primarily related to engineering, low-cost manufacturability, and clinical adoption. Ingestible devices carry a relatively low overall risk for patients and enable sub-chronic stimulation, thereby avoiding the complex regulatory landscape associated with implants. However, it is important to keep in mind that the digestive system is continuously sensing its lumen, and the presence of a device may inevitably alter the physiological state of the gut. To make a meaningful impact on patient care—whether for conditions affecting the gut or the brain—simplified devices must be urgently translated to humans. This will allow us to advance our understanding of how the human gut changes under such conditions before developing effective treatment strategies, including using the digestive system as an access point to the vagus or sacral nerves, which remains a fascinating translational pathway.

## Neuromodulation: novel modalities

A significant field of application for bioelectronics is the recording and modulation of neuronal activity, which has advanced considerably, with several technologies now integrated into clinical practice. Most of these devices record neuronal activity through local field potentials and/or employ electrical stimulation. Examples include neuroprostheses for speech decoding^96,97^, recording motor signals with stentrodes in patients with paralysis due to amyotrophic lateral sclerosis^98^ (Fig. 1), deep brain stimulation targeting the subthalamic nucleus^99^ (Fig. 1) or the lateral hypothalamus for movement disorders^100^, vagal nerve stimulation for a wide range of disorders^101^ (Fig. 1) as well as epidural electrical stimulation of the spinal cord (Fig. 1) or dorsal root ganglia for alleviating paralysis or pain^102–105^. In the periphery, integration of sensory information into prosthetic upper limbs has given amputees an unprecedented level of embodiment. Tactile and proprioceptive feedback can be delivered through intraneural electrodes^106–108^, while thermal sensation can be delivered to the surface of the residual limb at particular spots which elicit phantom sensation^109^. More recently, systems integrating both recording and stimulation systems have enabled closed-loop neuromodulation. Notable applications include motor cortex recordings to decode motor intentions, which can be paired with spinal cord stimulation for treating paralysis^110^, as well as the implantation of a deep brain sensing and stimulation device for biomarker-driven closed-loop treatment of psychiatric disorders including depression^111,112^. These closed-loop technologies continue to evolve to improve tissue compatibility, implant longevity, and recording and stimulation precision, as well as to integrate algorithms to facilitate adjustable, personalized neuromodulation.

Implantable neuromodulation strategies up to now primarily consist of electrical stimulation. However, despite its effectiveness across a number of disorders and many applications, it still faces certain limitations. While offering some degree of spatial and temporal targeting, it remains incredibly challenging to achieve cellular specificity as well as to inhibit neuronal activity, which is beneficial for certain clinical applications. With advancements in single-cell multi-omic techniques, we are now able to identify the diverse range of neuronal populations and understand their functional roles in neurological disorders, yet how these populations distinctly respond to electrical stimulation remains mostly unknown. Recent studies that model or measure the effects of different electrical stimulation paradigms on molecularly-defined neuronal populations have started to enhance our understanding of how specific neuronal populations can be targeted^113,114^. By fine-tuning electrical stimulation settings, cell specificity can be improved to some extent^99,102^, however, the similarities in neurophysiological properties between different neuronal populations limits cell-type targeting with electrical stimulation. Increasing cellular specificity remains a key goal in the field as minimizing the stimulation of undesired neuronal populations is required to mitigate off-target effects.

**Fig. 3:**
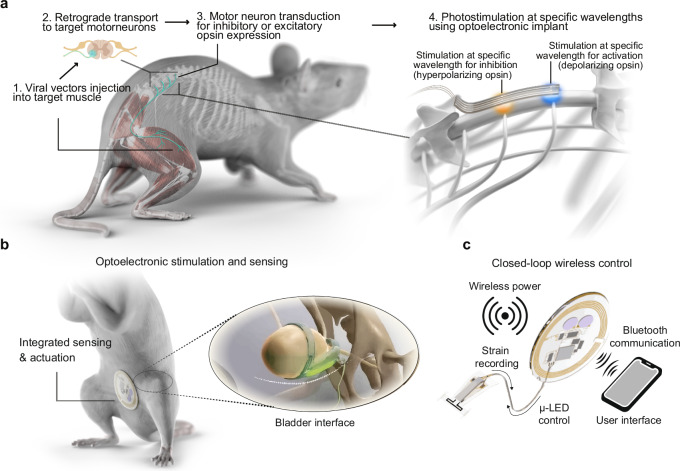
Optoelectronic devices for targeted closed loop neuromodulation. **a** Steps involved in the optogenetic neuromodulation of motor control, viral vectors are injected intramuscularly to retrogradely transduce and express excitatory or inhibitory opsins in spinal motor neurons, an implantable spinal LED array delivers photostimulation at specific wavelengths to activate or inhibit muscle activity. **b** An example of combined sensing and actuation modalities for closed-loop modulation of bladder activity. **c** Closed-loop wireless control of implantable device allows user to monitor and control neuromodulation.

Insights from single-cell multiomics have facilitated an exciting shift towards a new era of neuromodulation. A deepening of our understanding of the cellular and molecular mechanisms underlying diseases now enables the targeting of gene therapies to defined disease-related neuronal populations. Integrating these cell-type targeted gene therapies with advanced neuromodulation strategies, such as opto-, chemo-, and sonogenetics, aims to improve treatment efficacy while minimizing off-target effects. Notably, optogenetic tools offer unmatched spatiotemporal resolution, cellular specificity and bidirectional control of neuromodulation^115^, including efficient neuronal inhibition (Fig. 3). Over the past two decades, optogenetics has enabled extensive pre-clinical research uncovering the neural substrates of disease pathogenesis, and is increasingly being explored as a treatment approach for neurological disorders in small animal models, especially transgenic mice^116^.

Despite the advantages of optogenetics as a neuromodulation strategy, the field still faces major challenges to translate this approach to humans, as discussed in detail in Box 2. Ethical considerations and patient acceptance are key concerns, as optogenetics requires genetic modification of the target cells to express light-sensitive opsins combined with devices for photostimulation. Successful clinical translation depends on identifying appropriate indications/disorders, ensuring both safety and efficacy. Optogenetic therapy should be considered primarily for conditions with few existing treatments and where the potential benefits are high. Given these challenges, the most realistic near-term clinical applications of optogenetics are in the eye^117^, where its immune-privileged nature and the delivery barriers for both gene vectors and light are relatively low. Several clinical trials are testing optogenetic gene therapies for inherited retinal diseases such as retinitis pigmentosa^118^, and early results have demonstrated partial restoration of visual function in humans^119^. Unlike traditional retinal prosthetics, these approaches rely on the eye’s remaining neurons as the “hardware”, genetically converting light-insensitive cells (e.g., ganglion or bipolar cells) into new photoreceptors. This implant-free strategy offers a significant advantage in treating a wide range of blindness-causing mutations. By 2026, multiple programs are expected to reach Phase 2 or 3 clinical trials, including systems that eliminate the need for external headsets by using ultra-sensitive opsins or biochemical amplifiers. Therapies such as Nanoscope’s MCO-010 (channelrhodopsin delivered via AAV) have received both Fast Track and Orphan Drug designations from the FDA (NCT05417126, NCT04945772). Although patients may still require eye-mounted cameras or goggles and may experience low-resolution, monochromatic vision, for individuals with no light perception at all, regaining the ability to navigate obstacles or recognize large objects is life-changing. This represents a clear case in which optogenetics offers advantages that are not achievable with gene replacement or stem-cell therapies. Outside ophthalmology, optogenetic medicine is progressing more slowly and cautiously. One promising stratergy is the retrograde transduction and modulation of neurons at the interface of the peripheral and central nervous system, such as targeting spinal motor neurons to mitigate aberrant muscle activity (Fig. 3a), nociceptors to inhibit chronic pain or the sensory and motor neurons that control bladder function (Fig. 1). In the latter, the neurons innervating the bladder are genetically modified through virally delivered opsins, optoelectronic stimulation, gated by real-time biophysical sensing, could enable closed-loop control of bladder activity. Specifically, a soft, strain sensor monitors bladder distension and a micro-scale inorganic LED supports optogenetic control over neuronal activity and bladder function (Fig. 3b)^120^. A battery-free wireless communication module relays changes in strain to a customized user interface that provides real-time analysis of bladder volume and voiding frequency, as the basis for automated control to trigger optogenetic stimulation (Fig. 3c). This system uses light to control micurition and prevent symptoms of overactive bladder in response to the frequency and charateristics of volume changes.

Another example where optogenetic modulation is emerging as a key tool is to provide neuromodulation to the digestive system and the gut-brain-axis. Our digestive system performs highly complex functions that require a high level of signaling synchronization between sensory inputs, enteric neurons and pacemaker interstitial cells of Cajal. Thus, a standard electrical pacemaker with a fixed stimulation pattern has not been successful in restoring correct function, by gastric stimulation for example^121^. Advances in our understanding of gut function with recently developed ingestible and endoscopic devices show the need for improved localization of stimulation and personalization of the stimulation pattern, and ultimately the need for closed loop operation. It is thus likely that electrical stimulation is simply not sufficient and the activation and inhibition of specific neuronal types is required to mimic the highly orchestrated patterns of activity of the healthy gut, pointing to optogenetics as a potential solution. Optogenetic control of the intestine was first implemented in living anesthetized animals using a transparent window, showing that it is possible to control gut activity with light^122,123^. It has now been advanced to a wireless implantable solution that increased gut activity in freely moving mice^108^. More recently, a new optogenetic platform has been proposed to further advance our understanding of bi-directional gut-brain communication, where multifunctional sensing and stimulation microelectronic fibers implanted in the mice enabled wireless interfacing simultaneously with the brain and the gut in a freely moving mouse^124^. Another wireless optogenetic activation device implanted on the outside of the mouse colon induced an increased colonic function and allowed uncovering of an extrinsic brain-gut motor pathway via pelvic nerves^125^.

Demonstrating the effectiveness of optogenetic therapies in select disorders, outperforming existing treatments, will strengthen patient acceptance and facilitate further advancements in the field. The integration of optogenetics in the repertoire of clinical neuromodulation approaches would present a major step forward in offering alternative neuromodulation approaches with higher spatiotemporal and cellular precision.

Box 2 Challenges in the translation of optogenetics We are on the cusp of a new era of optogenetic therapeutic neuromodulation. Combining gene-therapy strategies to make cells photo-sensitive to light holds great promise for a number of therapeutic applications. A major advantage of optogenetics, besides its specificity, is its high spatio-temporal control. To maximize this property, strategies require a closed-loop system that measures physiological functions, detects abnormalities, and regulates photo-stimulation. Some examples of these systems have been mentioned in this perspective^102,120^. However, clinical implementation of optogenetics comes with several challenges, which we discuss here in detail.

The first challenge is in engineering opsins that through genetic modification can make tissue responsive to a desired wavelength of light. Various animal and microbial opsins are available with different functional properties, requiring carefully selection for each application. The most common opsins used in optogenetic research originate from phylogenetically distant species and therefore have the potential to evoke an innate or adaptive immune response in humans, posing a risk to diminish the long-term efficacy of these therapies^139–141^. Ongoing efforts aim to mitigate these risks by engineering microbial opsins with low immunogenicity by removing immunogenic epitopes to “humanize” them or include immune checkpoint molecules such as PD-L1/2 to mitigate the immune response^139,142^. Other approaches include re-purposing human opsins^143^ or designing artificial photocyclic animal opsins^144^, including chimeric human opsins to minimize and evade an immune response^145,146^. Recent advancements in the development of highly sensitive opsins^147,148^ and up-conversion nanoparticles which emit visible light following near-infrared excitation^149,150^ have improved the efficacy and translational potential of external light sources, which could facilitate regulatory approval for clinical translation.

The second challenge is linked to opsin delivery, which can be achieved through gene transfer therapy using safe and effective viral vectors (Fig. 3a). Gene therapies can be simple to administer, even with a single injection provided that sustained expression is achieved, but challenges remain in ensuring the gene delivery vehicle does not trigger host immune responses^141^, a risk that could potentially be mitigated through concomitant immunosuppressive strategies^151^. Gene therapies are steadily gaining acceptance as therapeutic interventions; there are currently 46 cell and gene therapy products approved by the FDA, with nine new cell and gene therapies approved for clinical translation by the FDA in 2024 alone, and an expectation that between 10-20 gene therapies will be approved annually from 2025^152^. Among all the delivery vehicles available, adeno-associated viral (AAV) vectors are the leading choice for the nervous system due to their safety, neuronal tropism and long-term therapeutic effect^153^. To date, three AAV gene therapies (non-optogenetic) have been approved by the FDA for use in the nervous system. Luxturna for inherited retinal dystrophy^154^, Zolgensma for spinal muscular atrophy^155^ and Kebilidi for Aromatic L-amino acid decarboxylase (AADC) deficiency^156^. Furthermore, optogenetics has already been successfully applied in human; subretinal injection of AAV2 expressing ChrimsonR, an excitatory opsin, partially restored vision in a patient with retinitis pigmentosa^119^. With advancements in single-cell transcriptomics, we are beginning to understand the diverse range of cell populations and their roles in different disorders^102,157^. Using this knowledge, we can engineer AAV vectors to target expression of opsins to specific neuronal populations that underlie disorders. This can be achieved by leveraging multi-omic datasets that combine single-cell RNA sequencing with ATAC sequencing to identify cell-type specific promoters, enhancer elements or unique cell-type defining transcripts to target expression of opsins to specific neuronal populations^158–160^, minimizing off-target effects and increasing safety and efficacy.

The third challenge involves hardware technology development, for the effective and safe photostimulation to activate opsins. Several approaches exist, each with distinct advantages and disadvantages. These include implantable micro-LED arrays, which can be shaped for specific organs, positioned close to target cells to decrease the required light intensity, and capable of targeting deep structures such as the spinal cord and bladder^120,161^ (Fig. 3a–b). Diodes can emit specific wavelengths, but integrating inherently hard diodes into soft implants is challenging. Other alternatives to deliver photostimulation are optic fibers, made of thin, flexible strands of glass or plastic, which are commonly used in research but have limited translational potential due to their rigid and bioincompatible properties. This has led to the development of hydrogel optic fibers, which are soft and stretchable and can integrate into soft tissues to transport light to deeper structures^162–164^. However, these still require connection to an implanted or external light source. Externally applied, transdermal or transcranial light sources can have good light penetration especially at longer wavelengths, offering a non-invasive solution to the target tissues that are superficial^147,165^. This approach is attractive because it avoids invasive procedures while remaining specific to targeted cells, although light penetration into deep tissues remains difficult.

The fourth challenge is the overall complexity and the lack of a clear regulatory strategy. From a regulatory perspective, the potential benefits of optogenetics are not in question; rather, the concern lies in the risks posed by gene transfer, medical devices, and their interaction^166^. While the integration of biology and technology offers tremendous scientific challenge and thus an opportunity for academic research, it also creates a significant regulatory and safety burden. Typically, the safest and ultimately most efficient strategy for advancing a complex medical device to first-in-human trials is a stepwise approach, where individual functions are tested separately in clinical trials. The FDA has experience regulating both gene therapies and implantable medical devices but has not yet approved an optogenetic therapy that combines the two. Therefore, one possible strategy is to demonstrate the efficacy of the gene therapy and the device independently. This approach is particularly useful in the U.S., where the risk assessment for a modified FDA-approved predicate device can leverage the original risk profile. Therefore, the medical device could be tested using a light-sensitve agent, or the gene therapy using a medical device that is already approved, such as those used in photodynamic cancer therapy. In Europe, an optogenetic therapy with an implanted device would be regulated both as a Gene Therapy Medicinal Product and as an active implantable device, necessitating coordinated evaluation between pharmaceutical and medical device regulators.

The final challenge is ensuring full ethical conduct of clinical trials. The idea of altering human neurons using foreign genes and then controlling them with light raises significant ethical questions. What are the long-term effects of introducing microbial opsins into human neurons? Could continuous or excessive illumination cause unforeseen damage? Might the presence of an implant affect a patient’s self-perception or autonomy? These issues are not merely hypothetical; they are actively being considered by researchers and ethicists as the first in-human optogenetic trials are being planned. A long list of potential applications for optogenetics exists—ranging from cardiac abnormalities such as arrhythmias^167^, to visual restoration^117,168,169^, hearing restoration^170^, epilepsy^171,172^, depression^173^, bladder control^120^ (Fig. 3b), neuropathic pain management^174,175^ and improving motor control in disorders such as essential tremor, Parkinson’s^176,177^ and spinal cord injury^161^ (Fig. 3a). Identifying the key applications, such as visual restoration, where patient benefits justify both genetic modification and device implantation needs to be clearly defined. Careful clinical trial planning will be essential, as highlighted by White et al.^166,178^. Unlike typical pilot safety and feasibility trials in healthy volunteers, this is not an option for optogenetics. It is therefore critical to start with a well-defined clinical indication and a specific patient population^179^. Ensuring truly informed consent is essential, as participants must fully understand the genetic basis of the therapy, the risks associated with neurosurgery, and the experimental status of the device component. Ethical considerations are paramount. Success in clinical trials is often measured by short-term outcomes, but in this case, long-term monitoring will be required, adding significant regulatory and budgetary burdens^178^. On one hand, incorporating AI and closed-loop operation will further increase regulatory complexity, but on the other hand it can facilitate substantially better outcomes of optogenetics and that additional complexity may be just what is needed to justify possible risks (Fig. 3c).

Overall, optogenetic modulation is a compelling alternative to existing therapeutic avenues for numerous disorders. However, as a new approach that involves both gene transfer and medical device components, ethical and regulatory frameworks need to evolve in parallel with the technology itself. Each incremental success—a patient regaining light perception, a regulatory agency authorizing a clinical trial—will help build confidence to pursue more complex applications, such as psychiatric disorders. If these barriers are overcome, optogenetics will fundamentally change how we interface with the nervous system through the precise language of light.

## Perspective on future developments

The future of in-body bioelectronics is promising. Their evolving capabilities and potential for multimodal applications make them ideally suited for achieving precision which is required in specific clinical contexts. Non-invasive approaches, while valuable, often lack the necessary precision for certain applications, further emphasizing the need of in-body systems. The trend for increased precision and personalized medicine also paves the way for new modalities, which will be increasingly seen as viable clinical alternatives offering precise control over biological functions by bioengineering specific cells to respond to various novel stimuli, such as photostimulation.

Reoccurring themes in the development of novel bioelectronics include innovative materials, the emergence of absorbable devices and the rising popularity of wireless technologies. An equally important aspect of future bioelectronics, particularly for multimodal systems integrating diagnostic and therapeutic actuation modalities, are closed-loop control systems, with real-time data analytics to interpret physiological cues and to trigger immediate functional modulation. AI driven autonomous algorithms will play an increasingly pivotal role in the evolution of these closed-loop systems (Box 3). Through their sensing capabilities, bioelectronic systems can capture subtle and/or rapid physiological changes, which are then processed by machine learning algorithms to detect abnormalities. The results can trigger therapeutic action, such as microfluidic drug delivery or electrical and optogenetic photostimulation, to correct the underlying issue. Compact, computationally efficient deep-learning algorithms embedded into processing power within the bioelectronic device can support biophysical pattern recognition for this purpose. A recent example of this strategy enables programmable closed-loop microfluidic pharmacology to suppress epileptic seizures based on real-time feedback from electroencephalograms^126^. These types of machine learning-driven bioelectronic technologies greatly enhances options in closed-loop control compared to traditional approaches, with promising implications for sophisticated, responsive, and personalized healthcare solutions^53,127–129^. In conclusion, the integration of advanced materials, novel forms of sensing and actuation, as well as the use of AI (Box 3) promise an exciting future for in-body bioelectronics, with the potential to redefine how we approach medical treatments and precision healthcare.

**Fig. 4:**
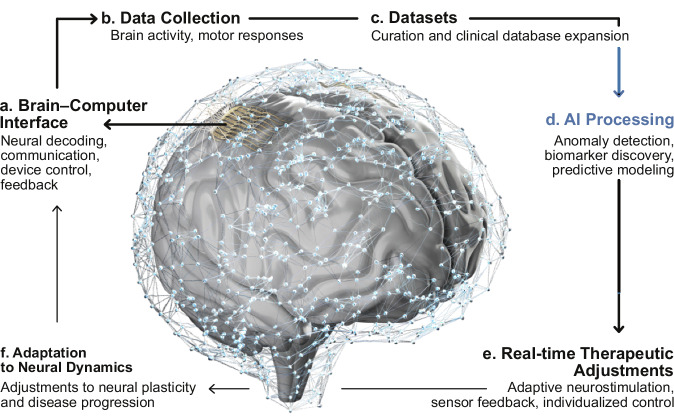
AI-enabled closed-loop neurotechnologies. **a** Neural decoding and encoding enable communication, device control, and sensory feedback. **b** Acquisition of brain activity and motor responses provides raw physiological signals. **c** Clinical data are curated and expanded into comprehensive databases that support model development. **d** Machine learning models perform anomaly detection, biomarker discovery, and predictive modeling. **e** Adaptive neurostimulation and sensor-driven feedback enable individualized control strategies. **f** Systems adjust continuously to neural plasticity and disease progression, ensuring long-term efficacy.

It has become clear throughout our Perspective, that multi-modal and interdisciplinary research is essential to finding solutions that address complex medical problems. However, the clinical translation of complex devices remains challenging—particularly when standards and medical device development controls are introduced only after a prototype has been developed. In the context of novel technologies and materials, regulatory considerations should be integrated as early as possible, ideally during the initial stages of pre-clinical work. Quality management systems play a critical role by imposing constraints that ensure the final solution is compatible with clinical use. When applied correctly, these constraints do not limit innovation; rather, they guide it toward novelty that can truly transform patient care. One of the main challenges is raising awareness of medical device development processes within academia so that new materials and technologies intended for medical applications can achieve their full transformative potential.

Box 3 AI for bioelectronic clinical application AI is increasingly central to clinical research, particularly in diagnostic decision support and closed-loop feedback systems. Machine learning algorithms can detect subtle anomalies across diverse data modalities—including electrophysiological signals, magnetic resonance imaging, and genomic profiles—thereby reducing inter-observer variability and supporting personalized treatment plans. For example, AI-driven analyses have enabled the identification of biomarkers associated with neurological disorders such as Parkinson’s disease, Alzheimer’s disease, and epilepsy^180–182^. While AI is becoming increasing capable, it needs to be explicit in the clinical community that AI remains as a clinical copilot, not an unaccountable autopilot. In our view, the progress will not come from the ever-increasing size of the models but more from constrained, auditable systems with quantifiable uncertainty and predictable outcome, which are designed around clinical workflows and endpoints.

Beyond diagnosis, closed-loop neurostimulation systems leverage AI to process real-time physiological signals, such as brain activity or motor responses, and adapt stimulation parameters dynamically (Fig. 4). Models trained on large-scale neural datasets can predict optimal response patterns for individual patients, maintaining therapeutic efficacy while mitigating adverse effects. This adaptability is particularly valuable as neural plasticity and disease progression alter patient responses over time. Reinforcement learning frameworks further enhance these systems by allowing treatment strategies to evolve autonomously, which must be bound by design standards such as hard safety constraints, clinician-overridable policies, and conservative update rules. These principles naturally extend to brain–computer interfaces (BCIs) with representative applications in communication^96,183^, external devices control^110,184,185^, and sensory feedback^106,186^, where decoding improvements will play a pivotal role clinically but only when paired with reliability, calibration, and clear failure modes.

A central bottleneck inherent to the development of higher-density interfaces is data transmission throughput: more channels require significantly higher bandwidth, power and reliability, which concerns the scalability of the system in clinical settings outside of a laboratory. Clinical deployment will naturally rely on edge computation rather than indiscriminate transmission of raw data. In this view, next-generation platforms should integrate sensing, on-device processing, and communication within a co-designed pipeline that supports adaptive sampling, local feature extraction and representation compression, and event-driven transmission, prioritizing clinically relevant information extracted from full-resolution signals. Such approaches will reduce end-to-end latency in closed-loop systems and enhance privacy by only sending processed data while retaining sensitive data on device reserved for clinical necessity.

The integration of AI into clinical research is not without challenges. Ethical and long-term safety considerations require a more balanced framing than risk-benefit assessment alone. Patient privacy, algorithmic bias, and accountability remain fundamental^187^. AI models can perpetuate biases present in training data, potentially leading to disparities in diagnosis or treatment outcomes among underrepresented groups. Moreover, the decisions made in critical interventions such as closed-loop neurostimulation or personalized medicine may raise questions about accountability in cases of adverse outcomes. In addition, as data-driven models are sensitive to distributional shifts, known as concept drift, there is a need for robust validation studies to ensure the reliability and safety of AI-powered tools^188^. Even with these challenges, the potential benefits of AI are likely to outweigh the risks, providing higher quality diagnosis and treatment options to patients. Finally, long-term safety should be addressed as a primary design and translational consideration.
